# Wood’s Medical and Surgical Monographs

**Published:** 1890-03

**Authors:** 


**Wood's Medical and Surgical Monographs.** Vol. IV. No. 3. December, 1889. Published monthly. $10.00 a year. Single copies, $1.00. New York: William Wood & Co., 56 and 58 Lafayette place.

This is the final number of this interesting and valuable series of publications for the year 1889. We are gratified to learn that the publishers will continue the regular monthly issuance of similar books during 1890.

The present number contains the following titles: A Practical Treatise on Baldness, by George T. Jackson, M. D.; The Sphere, Rights, and Obligations of Medical Experts, by James J. O'Dea, M. D.; The Pathology and Treatment of Scarlet Fever, by Dr. H. Von Ziemssen ; Pathology and Treatment of Ringworm, by George Thin, M. D.; Notes on Dental Surgery, by J. Smith, M. D., L. L. D.; On Sounding for Gall-Stones, and the Extrusion of Gall-Stones by External Manipulation, by Dr. J. Harley, F. R. S.

The Monographs of Drs. Thin and Harley are illustrated with numerous wood-cuts. The title of Dr. Ziemssen's paper was omitted from the cover page, but it is one of the best in this number. A complete index of Vol. IV. accompanies No. 3, which is a book full of interest.

